# The Impact of Critical Listening and Critical Reading on Critical Thinking

**DOI:** 10.3390/bs15010034

**Published:** 2025-01-01

**Authors:** Yasemin Baki

**Affiliations:** Department of Turkish and Social Sciences, Faculty of Education, Recep Tayyip Erdoğan University, 53200 Rize, Turkey; yasemin.baki@erdogan.edu.tr

**Keywords:** critical listening, critical reading, critical thinking

## Abstract

Critical listening, critical reading, and critical thinking are three closely related cognitive skills that aim to evaluate information with an analytical and questioning approach. Critical listening and critical reading, which are receptive language skills, represent the application of critical thinking in different contexts. Critical thinking, which is a productive language skill, provides a framework for these two receptive language skills and enables the evaluation of the accuracy of information accessed through critical listening and critical reading, analyzing different perspectives and making inferences to reach correct conclusions. These two skills support the development of critical thinking skills and contribute to individuals gaining deeper understanding based on the perspective of knowledge. This study aims to determine the relationships between critical listening, critical reading, and critical thinking, the effects of these variables on each other, and the explanation ratios. The study group of this study was determined through simple random sampling, one of the random sampling methods. The participants consisted of 201 teacher candidates studying in the Department of Turkish Language Teaching at a university in the north of Türkiye. The Critical Listening Scale, Critical Thinking Attitude Scale, Critical Reading Self-Efficacy Perception Scale, and a personal information form were used to collect research data. The data collected in the research were analyzed using structural equation modeling via AMOS 22.0. As a result of the research, it was determined that all hypothesis models established based on the relevant literature were valid. Two of the three hypotheses regarding the theoretical model were supported by the data, and one hypothesis was rejected. Critical listening has a direct high level effect on critical thinking and predicts it at a significant level. Critical listening has a direct high-level effect on critical reading and predicts it at a significant level, while explaining 65% of the total variance related to critical reading. The effect of critical reading on critical thinking is insignificant and does not predict critical thinking at a significant level. In the theoretical model created the effect of critical reading on critical thinking is insignificant, but these two variables explain 85% of the variance related to critical thinking. As a result of the research, it can be said that the main predictor of critical thinking is critical listening, and that critical reading and critical thinking develop depending on the development of critical listening.

## 1. Introduction

Twenty-first century skills are the skills that individuals must have to cope with the innovations brought about by Industry 4.0, the fourth industrial revolution, and to adapt to life in this century (Sullivan et al., 2020). These skills, which individuals must possess and constantly develop to be successful in daily and business life, are classified in many ways due to the complexity and ever-changing structure of this century (Lai & Viering, 2012). Twenty-first century skills, which include many skills and abilities that are not easy to define, have been defined by many institutions and organizations such as the ISTE, NCREL, ATC21S, EU, OECD, and P21 to train qualified workforce in this century, and various classifications have been put forward for these skills (Hamarat, 2019; Ledward & Hirata, 2011). Although these classifications differ in categories and emphases, the skills that are commonly emphasized in P21 are learning and renewal skills (P21, 2019), in the OECD 2030 Future of Education and Skills Report (OECD, 2019) within cognitive and metacognitive skills, and in the ATC21S Report (ATC21S, 2010) within thinking skills; critical thinking, creative thinking, problem solving and decision-making skills (Care et al., 2010; González-Perez & Ramírez-Montoya, 2022; Kiraz, 2022; WEF, 2016).

One of the skills that has come to the forefront in the 21st century and is gaining importance day by day is critical thinking (Willingham, 2019). The diversity, amount, and speed of dissemination and access opportunities offered by information, communication, and educational technologies with the innovations revealed by the digital information age and the internet industry are effective factors in the prominence of this skill (R. Gupta, 2021; Tiemann & Annaggar, 2020). Because this transformation provides faster and easier access to information, it also rapidly increases the possibility of encountering incorrect, incomplete, or misleading information (Castells, 2004; Schwab, 2016). In addition, in today’s complex and rapidly changing world where information pollution is increasing, it has become a necessity on both personal and social levels for individuals who are stuck in the middle of information masses to be able to access accurate information to sustain their lives (Herodotou et al., 2019; Shavelson et al., 2019). For individuals to access accurate information, it is necessary to critically examine the source, accuracy, logical consistency, and reliability of the information accessed (B. J. Smith, 2022).

For individuals to be able to select, analyze, and evaluate information in this environment; to turn information into a discovery force in their lives; to solve complex problems; and to make critical decisions on their own, they need to approach information with a critical perspective and use their independent thinking skills (Elder & Paul, 2020; Ennis, 2018; Facione, 2011). In this context, critical thinking stands out as a skill that expresses the individual’s ability to make informed, conscious, and logical decisions today (Butera et al., 2014) and is considered one of the vital thinking skills that individuals must have to survive in this century (Lin & Luk, 2015).

Critical thinking is among the skills that individuals need to have to increase their knowledge capacity and choose the right information, as well as to structure information and produce new information (Zoller et al., 2000). In the 21st century, with the transition from an industrial society to an information society, the understanding of the nature of information has changed with the change in information technologies. With this change, the understanding of individuals being loaded with information has been abandoned, and instead, studies aimed at increasing the capacity to produce information have been brought to the fore (Gültekin, 2020). This change in understanding regarding the structuring of information has also restructured the competencies that individuals must have, and instead of individuals who acquire information, individuals who can structure information have been brought to the fore (Niess, 2005). Thus, today’s individuals have met a new age called the conceptual age, where those who produce meaning can exist (R. Gupta, 2021; Pink, 2006). For individuals to produce new meaning, that is, new thoughts, they need to use their thinking skills as well as their basic language skills, which function as channels for obtaining and structuring information (Jones et al., 2020; Vygotsky, 2018).

Basic language skills, consisting of listening, reading, speaking, and writing skills, are integrated and spiral mechanisms, and the development of these skills is coordinated (Sadiku, 2015). The development and skillful use of language skills, which serve to acquire information through two basic functions, receptive (listening and reading) and productive (speaking and writing), is a functional process that enables the assimilation of information from different sources and channels and its transfer to life (Jewett, 2007). For this process to operate effectively, the combined development of language skills and thinking skills, which are the output of these skills, is necessary (Aghajani & Gholamrezapour, 2019).

Today, access to information and the increase in the speed of dissemination of information have also revealed new text structures (Papadopoulou et al., 2018). As texts become increasingly complex, multi-layered text structures and the information, ideas, and evidence they reveal cannot be assimilated only through reading, listening, and speaking skills, but these skills must be supported by critical skills (Dewati, 2020; Zin et al., 2014).

The combined use of critical reading and critical listening skills, which are receptive language skills, has a key role in this period when accessing accurate information and structuring information is increasingly important (M. Wallace, 2021). These two receptive language skills represent the application of critical thinking in different contexts (Cruz et al., 2017; Erkek & Batur, 2020). In this process, critical reading goes beyond the meaning of a text, enables the text to be questioned and judged, evaluates the content and context of the text from different perspectives, and makes inferences about the text (R. Paul & Elder, 2019). Similarly, critical listening removes the individual from the role of a passive receiver and allows for him/her to analyze the content he/she listens to, question its accuracy, and make contextual evaluations instead of passively consuming it (Brownell, 2015). These two skills support the development of critical thinking skills by encouraging individuals to approach information critically, make in-depth analysis, question information, evaluate opposing views and develop a more conscious thinking process, and contribute to individuals gaining deeper understanding based on information and producing new knowledge (Kamçı, 2024).

Associating critical reading and critical listening processes with critical thinking not only improves individuals’ individual learning processes but also enables them to participate more consciously at a social level. For example, while individuals can notice hidden meanings and prejudices in written content through critical reading (Shokouhi & Latifi, 2019), they can become more resistant to misinformation through critical listening skills (Facione, 2011; Fisher, 2014). Thus, it contributes to the critical thinking process and enables the production of better, more accurate, and more beautiful thought products from what is read and listened to. Therefore, the effective use of these skills is extremely important for the development of individuals’ critical thinking skills. In addition, since information comes from multiple sources today, it is necessary to use critical thinking, critical reading, and critical listening skills together and effectively to analyze, evaluate, and produce information from these different information channels (Mamman-Muhammad, 2018; Wechsler et al., 2018). In summary, critical listening, critical reading, and critical thinking, which are interrelated cognitive skills that aim to evaluate information with an analytical and questioning approach, are among the basic skills that an individual must have in accessing accurate information and creating new knowledge (Cruz et al., 2017; Kamçı, 2024).

### 1.1. Theoretical Perspective

In this section, firstly, the theoretical foundations of the variables in this research are emphasized, and then the theoretical relationships between the variables are examined.

#### 1.1.1. Critical Thinking

The word critical is derived from the Greek words “kritikos” (insightful judgment, ruling) and “criterion” (standards) and passed into Latin as “criticus”. Its English equivalent is “critical”, meaning evaluation, judgment, distinction, and in Turkish, it means making a judgment about the value of something (Cifçi, 2006; Kaya, 1997). Critical thinking, which focuses on evaluating something with its good and bad aspects and deciding, can be defined as the process of creating standards to objectively determine the true value of something and reaching a decision according to these standards (Ennis, 2011; R. Paul & Elder, 2016). Critical thinking, which actively uses strategic thinking in this process (Dinsmore & Fryer, 2023), starts with asking questions (R. W. Paul, 2012). It is a logical and reflective thinking skill (McPeck, 2016; Nosich, 2016) that enables us to question information to test its accuracy (Bowell & Camp, 2018; Butera et al., 2014) and analyze it (Lovelace et al., 2016), to select the correct information by evaluating sources and evidence (Rousseau, 2012), and to make decisions (Rousseau, 2012; Rudd, 2007; G. F. Smith, 2014).

Critical thinking, one of the higher-order thinking skills, does not consist of a single skill or sequential skill sets, so there are various approaches to classifying critical thinking skills (Facione, 2011; Nosich, 2016; R. Paul & Elder, 2019). Facione (2011) lists these skills as analysis, interpretation, inference, explanation, and evaluation; Halpern (2014) lists them as drawing conclusions, analyzing, testing hypotheses, seeing possibilities, decision making, problem-solving, and creative thinking. Critical thinking, which is a multi-dimensional structure, uses metacognitive skills such as assimilating, processing information, and evaluating ideas, as well as self-evaluation, monitoring, and correction (Ennis, 2018; Taglieber, 2000).

At the center of critical thinking, which encompasses multiple skills and abilities (Shalova, 2015), lies the effort to reach real and accurate information with the power of thinking (Erkek & Batur, 2020). In this way, instead of blindly believing the information that others try to make us believe, it aims to develop acceptable objective criteria for beliefs and behaviors to distinguish right from wrong (R. Paul & Elder, 2016; Willingham, 2019). Because this thinking skill argues that there is no single truth based on the idea that knowledge changes according to place and time, and therefore rejects stereotyped judgments (Elder & Paul, 2020; Fisher, 2011, 2014). For this reason, it aims to reject one-sided perspectives and perceive events from multiple perspectives, thus developing unlimited thinking (McPeck, 2016). This aspect transforms critical thinking into a vital key skill that an individual must use effectively to think quickly, choose the most accurate one, and make the most accurate decision among the multiple alternatives presented in all areas of life that are constantly evolving and changing (Ferrari-Bridgers et al., 2017a). Individuals who can think critically transform knowledge into an exploratory force in their lives (Lin & Luk, 2015). Thus, it becomes a life skill that facilitates access to accurate information while acting as a shield and filter against incorrect, incomplete, and false information (R. Paul & Elder, 2019).

#### 1.1.2. Critical Listening

Critical listening is an advanced, intensive listening activity (Ediger, 2015) in which cognitive and metacognitive skills are used together to understand and evaluate what is being listened to from all aspects (Trace, 2013). This type of listening consists of the stages of organizing the ideas being listened to, analyzing the connections between them, and determining the level of importance of the ideas (R. Wallace, 2013; Trace, 2013). In this process, it encourages the listener to listen, question, evaluate, and decide on the information or ideas presented (Basyoni & Medd, 2023). Thus, it identifies subjective expressions, orientations, propaganda, and misconceptions in what is being listened to; questions and evaluates what is being listened to from an impartial perspective with its positive and negative aspects (Ferrari-Bridgers et al., 2017b); and ensures that the adequacy of what is being listened to is determined (Hyytinen et al., 2021). It uses an impartial perspective to evaluate the accuracy and reliability of what is being listened to (Corey, 2016). In this way, by instilling in the listener the habit of asking questions instead of blind criticism, the listener is enabled to find the truth (Doğan, 2011; Hiner, 2016) and to make a judgment by reaching logical conclusions with strong reasons (Basyoni & Medd, 2023; Sastromiharjo et al., 2020).

The main purpose of critical listening, which is one of the skills within the scope of listening for information, is to reach the right conclusions by making correct judgments based on what is listened to (Ediger, 2015; Trace, 2013). Today, due to the influence of technology, the individual is exposed to listening elements from multiple sources, and due to the fact that he/she comes across a multitude of propaganda, persuasive speeches, visual elements, and videos on social platforms in his/her daily life, using critical listening skills to effectively collect, evaluate, and use the right information has become a basic need (Aslan, 2021; Din, 2020). This skill acts as a shield against the individual doing wrong things in his/her daily life and as a compass to reach the truth, thus protecting him/her from mistakes (Bowell & Camp, 2018). Since critical listening is used not only in making sense of information but also emotions and experiences, it is one of the skills that contribute to the individual’s life in a social and cultural sense (Elmosnino, 2022). This skill enables one to approach what one listens to/watches from an impartial and objective perspective, to stay away from prejudices and generalizations, and to be fair and respectful to ideas (R. Paul & Elder, 2016).

#### 1.1.3. Critical Reading

Based on Freire’s Critical Theory and pedagogical practices, critical reading is based on the idea that understanding what is read is not enough to construct knowledge with the changing perception of reading in the 20th century, and that the individual must have critical reading skills for this (Hoody, 2008). Critical reading is a high level of functional reading in which metacognitive skills are used to break down the text into its parts and reassemble these parts in a coherent way to clarify information, to establish evaluation standards and to evaluate the accuracy of ideas according to these standards (Goatly, 2013; Spears & Spears, 2006).

Critical reading examines what is read in a three-stage process: understanding, questioning, and evaluating (Kress, 2010). In the critical reading process, various subcomponents related to understanding are used to understand the text in depth. These components are defining the topic; determining the thesis and main idea in the text; paying attention to key concepts; being able to identify the chronological order; finding the author’s point of view; distinguishing facts and opinions; noticing prejudices, stereotypes, and assumptions; noticing consistencies and contradictions in the text; finding old information and beliefs and comparing them with prior knowledge; comparing the author’s views with one’s own views; interpreting; drawing conclusions; and evaluating (Pirozzi, 2003; Flemming, 2011; Demir, 2017).

In this reading process, it is necessary to reveal the author’s intention by separating the text into its components with a selective approach (Cervetti et al., 2001; Knott, 2005), and to interpret and evaluate the text for a high level of understanding of the reading material. References are used to go beyond what is explicitly stated in the text, gaps in the text are filled, the subtleties of the meaning in the text are reached to discover the depths of the meaning in the text and the implicit meaning is revealed, and logical conclusions are reached by evaluating the information accessed (Pirozzi, 2003; Shokouhi & Latifi, 2019). In addition, while reading, the focus is on making inferences to predict, test hypotheses, and avoid judging and making judgments about that information until definitive evidence is obtained (Spears & Spears, 2006). For this purpose, in addition to understanding and analyzing the text, it is ensured that the sources are examined; the author’s purpose is recognized; the author’s propaganda tools, claims, and arguments are identified; and the author’s ideas and fantasies are distinguished from the truth (M. Wallace, 2021), because with the equipment they have, the reader evaluates the text in its entirety by realizing not only what was written, how it was written, and to whom it was written, but also why it was written.

In this process, critical reading focuses on reconstructing meaning rather than comprehending it by enabling the reader to enter a rich and interactive dialogue with the text (Özdemir, 2011). In this way, it takes the reader beyond being a passive receiver and beyond what the author says (Hoody, 2008), because critical reading argues that all texts are limited by the author’s perspective, and therefore every text should be approached with skepticism, focusing on the idea that what really matters is to go beyond the author’s perspective and produce new meaning (Kaur, 2013; Kress, 2010). In this way, the reader is enabled to combine what they have learned through reading skills with their own knowledge and experience and reconstruct them, thus producing better, more beautiful, and more accurate mental products (Cifçi, 2006).

#### 1.1.4. The Relationship Between Critical Listening and Critical Thinking

Critical listening and critical thinking are related skills because they have common sub-skills (Smialek & Boburka, 2006). In this relationship, critical listening, which is considered the fundamental part of critical thinking (Hyytinen et al., 2021; Kemiksiz, 2015), is the application of critical thinking skills in verbal communication (Aslan, 2021).

In critical listening, since it is essential to analyze, evaluate, and reorganize the information presented in depth rather than comprehending it, it is aimed at understanding what is being listened to at a high level (El Gendy, 2020; Floyd & Clements, 2005; Gunawan et al., 2023). Critical listening, which is also defined as the ability to understand and evaluate what is being listened to (Floyd & Clements, 2005), offers the opportunity to develop critical thinking skills with skills such as high-level thinking, reasoning, and rationalization used in structuring information (Gunawan et al., 2023; Wuryaningrum et al., 2022). In other words, it puts what is being listened to through a critical listening filter and reconstructs what it passes through this filter with critical thinking skills, thus enabling the production of new and correct thoughts. Thus, it contributes to the development of unlimited thinking skills by improving individuals’ thinking capacities (Tubail, 2015).

However, it is known that critical thinking skills are not sufficiently developed (Harefa, 2024) due to the lack of importance given to critical listening in the education process (Bell, 2018; Welch & Mickelson, 2020). Kamçı’s (2024) research determined that critical listening is a significant predictor of critical thinking. In addition, studies in the literature emphasize the need to develop critical listening skills for the development of critical thinking skills (Aslan, 2021; Smialek & Boburka, 2006). Based on these reasons, it can be said that critical listening should be given the necessary importance for the development of critical thinking and that coordinated action should be taken in the development of these skills.

#### 1.1.5. The Relationship Between Critical Reading and Critical Thinking

Nowadays, with the influence of media and social media, readers need to actively use their critical thinking skills as well as their critical reading skills (Winter, 2018) to access accurate information within vast information masses and to create their own truth (C. J. Lee, 2016). Individuals need to actively use critical reading and critical thinking to separate the interesting and useful parts from the texts they read and to read selectively (Flemming, 2011).

In the critical reading process, the reader is provided with the habit of asking questions about the text, thinking about the subject of the text, evaluating the subject with its positive and negative aspects, and finding his/her own truth (Spears & Spears, 2006). While reading the text critically, the reader evaluates the text in terms of many elements such as logical consistency, plan, order, and tone of the text by moving from the surface structure to a deeper structure (C. Wallace, 2003) and uses more than one level of thought interactively at the same time in this process (Taglieber, 2000). In this interactive process, while a conclusion is ideally reached through thinking, questioning, and evaluation, critical, analytical, and reflective thinking processes are actively operated, going beyond cognitive skills, such as reading comprehension (Wilson, 2016).

Critical reading is considered the first and most important step of critical thinking and is considered one of the skills necessary to provide readers with a critical perspective (Sadioğlu & Bilgin, 2008; Karadeniz, 2014). This is because, for a reader to evaluate a text critically, they must first have critical reading skills, that is, they must be able to examine the text with a judgmental and questioning approach, and for this, they must use analysis, synthesis, and evaluation skills (Basmaz & Kutlu, 2021). When these skills are examined, they are skills that are actively used in the critical thinking process. In other words, critical reading and critical thinking are skills that include common skills and are intertwined (Kurland, 2000). For this reason, it can be said that the use of critical thinking skills is also necessary for a text to be read critically (Zin et al., 2014). Because when analyzing a text, the text is questioned with critical and analytical thinking processes, that is, with the power of thought, and the information in the text is transformed into wisdom (Mamman-Muhammad, 2018; Talebi & Marzban, 2015). Based on these reasons, it can be said that critical reading and critical thinking skills, which are also at the focal point of the education system today, need to be developed systematically and in a coordinated manner (Mbato, 2019; Moore, 2011).

Various studies have been conducted in the literature examining the relationship between critical reading and critical thinking at different levels of education and drawing attention to the importance of this issue (Abdel Halim, 2011; Larsson, 2017; Kamçı, 2024; Y. H. Lee, 2015; Mayfield, 2014; Medina & Pilonieta, 2006; Özensoy, 2011). Based on these studies, it can be said that critical thinking and critical reading skills are interrelated skills and that the development of critical reading is necessary for the development of critical thinking.

#### 1.1.6. The Relationship Between Critical Listening and Critical Reading

Critical listening and critical reading skills are defined as interrelated skills (Mamman-Muhammad, 2018). It is predicted that the interactive use of these skills, which have common sub-skills, will make positive contributions to the development of both skills. In the literature, it has been revealed by various studies that there is a positive relationship between critical reading and listening/watching (Arono, 2015; Arslan, 2022), that listening materials strengthen critical listening (Puspita & Amelia, 2020), and that critical listening activities improve comprehension of what is listened to (Azizoğlu, 2020; Çarkıt, 2018). In the study conducted by Kamçı (2024) with Turkish and English teacher candidates, it was determined that critical reading is a positive predictor of critical listening. When the relevant studies are examined, it is seen that the focus is on the effect of critical reading on critical listening, the effect of critical reading on listening skills, or the effect of critical listening on listening skills. However, although no research has been found on the effect of critical listening on critical reading, it is predicted that critical listening and critical reading are related concepts (Mamman-Muhammad, 2018). It is predicted that examining the relationship between these two variables will contribute to the development of both skills.

#### 1.1.7. The Relationship Between Critical Reading, Critical Listening, and Critical Thinking

Critical thinking is a skill acquired through social means and education (C. Wallace, 2005). One of the basic missions of educational institutions is to raise modern individuals who can distinguish right from wrong and shape their future in the most accurate way by using these skills (Besoluk & Önder, 2010). For learners to cope with the challenges of this century, they need to analyze expressions, be aware of unexpressed thoughts and prejudices, determine different expressions of thought, question the reliability of sources by considering existing prejudices, and reach the most accurate decision with the evaluations made (Elder & Paul, 2020; B. J. Smith, 2022). For this, learners need to actively use critical thinking skills so that they can trust themselves but act cautiously and think in the most accurate way (Redhana, 2021). Critical thinking, which has been defined as one of the basic skills in educational programs due to its importance (Bailin & Siegel, 2003; Hafni et al., 2020), has also been accepted as one of the basic components of higher education (Baillargeon, 2016; Gray, 2016; Shavelson et al., 2019).

Although various methods and techniques are used to develop critical thinking skills, it has been revealed that the desired level of development of these skills has not been reached (Ardini et al., 2020), university education is inadequate in the development of critical thinking skills (G. Gupta, 2005; Zohar & Schwartzer, 2005), and education faculties do not provide sufficient contribution to the development of critical thinking skills of prospective teachers (Besoluk & Önder, 2010). Since the quality of education is accepted as equivalent to the quality of the teacher (Stacey, 2010), ensuring the development of critical listening and critical reading skills for the development of critical thinking skills in teacher education has a critical function in terms of both the professional development of teachers and increasing the quality of students and education (MEB, ÖYEGM, 2008; Kamçı, 2024).

Despite the need for critical listening for the development of critical thinking skills (Aslan, 2021; Smialek & Boburka, 2006), the limited number of strategies and educational materials for critical listening (Basyoni et al., 2020) and the lack of importance given to critical listening negatively affect the development of critical listening skills (Bell, 2018; Welch & Mickelson, 2020). However, individuals need to listen critically and produce new ideas using critical thinking skills to evaluate the content of the input conveyed through oral language. Yet, deficiencies in critical listening negatively affect the development of critical thinking (Aslan, 2021).

Critical listening, which has an effective power in the development of language skills as well as thinking skills (Gunawan et al., 2023), is also a skill related to critical reading (Mamman-Muhammad, 2018). In Kamçı’s (2024) research, it was determined that critical reading was a positive predictor of critical listening. On the other hand, no research examining the effect of critical listening on critical reading in the relationship between these two skills, which are receptive language skills, was found. In addition, the power revealed by critical reading and critical thinking, which are common skills and intertwined skills (Kurland, 2000), is at the focal point of the education system today (Moore, 2011; Stacey, 2010), and individuals need to have critical reading skills to think critically (Kamçı, 2024; C. J. Lee, 2016; Lewis et al., 2006). These skills, which are seen as educational goals in many societies around the world, have also been accepted as the key to success in higher education (Davies & Barnett, 2015; Khamkhong, 2018).

As a result of the relevant literature review, the relationships between critical listening, critical thinking, and critical reading, which have an active role in the education process, have been revealed and presented with the support of various views and research results that these skills interact with each other. However, gaining a critical perspective, which is increasingly important today, is too comprehensive a concept to be addressed in a single dimension. This is because individuals who appeal to different sense organs and are exposed to a constant flow of information need to evaluate this information with their thinking power and access the most accurate information (Jones et al., 2020; Zarzavatçıoğlu, 2023). For this, in addition to the need to use different language skills such as listening and reading collaboratively (Onan, 2010), it is also necessary to use these skills together in a critical way (Mamman-Muhammad, 2018).

Critical reading and critical listening, which are among the cognitive strategies of critical thinking, interact, and the development of these three skills should be considered together (M. Wallace, 2021). In the research conducted by Kamçı (2024) with prospective teachers, the relationship between these three skills was examined, and it was determined that critical reading was a significant positive predictor of critical listening and critical thinking. In addition, theoretical research was conducted by Mamman-Muhammad (2018) including views on the impact of these three variables on each other and the importance of these skills. In addition, Erkek and Batur (2020) created a critical listening acquisition list by using these three skills, and Dewati (2020) examined the effect of critical thinking skills on reading and listening skills. Based on these studies, it can be said that there are a limited number of dimensional level studies that reveal the use of these three variables together and that the majority of these studies provide theoretical information about the use of these three variables together. Only in Kamçı’s (2024) study was the relationship between these variables examined with structural equation modeling, but in that study, although the focus was on critical thinking standards, critical listening, reading, speaking, and writing skills were evaluated together. In this study, the focus was on the relationship between receptive language skills and productive skills in the theoretical model created based on the literature. In this context, the relationship between receptive language skills, critical reading, critical listening, and critical thinking, which is a productive skill, and their effects on critical thinking skills were examined with structural equation modeling, and the effects of these variables on each other and their explanation rates were examined. The focal point in the creation of the research model was the relationship between basic language skills and thinking skills (Aksan, 2007; Vygotsky, 2018). Since language skills are both the source and carrier of thinking skills (Yalçın, 2010), the focus was on the relationship between language and thinking skills in this study.

Thinking, which is a production made because of the actions of the mind, is the process that allows for an individual to process the information received and create meaning (Hotaman, 2008). The aim of the effective use of language skills is to reach accurate and complete thoughts and to create meaning (Yalçın & Şengül, 2007). To establish an effective relationship between language and thought, it is necessary for the language to be transformed into a good template for the thought to be conveyed (Vygotsky, 2018). In this context, basic language skills are divided into two in the literature as comprehension skills (listening and reading) and expression skills (speaking and writing) (Karatay, 2014). The basic language skills that undertake the receptive and leading role in the creation of meaning through thought are comprehension skills.

In this study, the relationship between critical language and thinking skills was examined, and the relationship between critical listening and critical reading (M. Wallace, 2021), which are receptive skills, and critical thinking (Gilstrap & Dupree, 2008), which are productive skills, was excluded from the scope of this research, while critical speaking and critical writing, which are productive language skills, were excluded from the scope of this research. The reason why critical reading and critical listening were selected among the language skills in the model is that both are receptive language skills and follow the mental processes that progress from the surface structure to the deep structure in the creation of meaning (Onan, 2015). The reason why critical listening was selected as the starting point of this model is that it is the first language skill to develop in the individual and is used at a rate of almost half in daily life (Arıcı & Taşkın, 2019; Pinnell & Jagger, 2003). In establishing the relationship between critical reading and critical thinking, the fact that critical reading and critical thinking use common cognitive skills is that they are skills that interact with each other and cannot be considered separately (Cotuksöken, 2011; Kamçı, 2024; Karadeniz, 2014; Kurland, 2000).

In critical reading, information and ideas in the text are discovered, while in critical thinking, information and ideas are evaluated to decide what to believe from the information in the text (Kurland, 2000). In addition to the mutual relationships of critical reading and critical listening with critical thinking (Kurland, 2000; Smialek & Boburka, 2006), critical reading and critical listening are the first steps of critical thinking and are the application of critical thinking in the receptive language dimension (M. Wallace, 2021). Critical thinking, which is a productive skill, sometimes provides a framework for these two receptive language skills and allows evaluating the accuracy of the information accessed through critical listening and critical reading, analyzing different perspectives, and making inferences to reach the right conclusions (Cruz et al., 2017; Erkek & Batur, 2020). Sometimes, the information received through these two skills supports the development of critical thinking skills through critical listening and critical reading processes, contributing to individuals gaining deeper understandings based on knowledge and producing new ideas (Kamçı, 2024; Wechsler et al., 2018). In summary, the combined use of these three closely related skills provides an opportunity to achieve significant gains in terms of the development of critical skills (Mamman-Muhammad, 2018; Wechsler et al., 2018).

Based on the relevant literature review, this research model, which reveals the relationship between critical listening, critical reading, and critical thinking, aims to reveal the relationship between the information perceived through receptive language skills and the production created through thought and the interaction between them. It is anticipated that the results of this research will provide various contributions to the organization of learning-teaching activities by revealing the effect of critical listening and critical reading in teaching activities to be carried out for the development of critical thinking skills. It is also thought that it can help develop new teaching methods and strategies regarding the importance of these skills. In addition, it is expected to contribute to the literature in terms of strengthening the theoretical framework and deepening the conceptual framework with the concrete data presented regarding the relationship between critical reading, critical listening, and critical thinking and the effects of these variables on each other. In addition, since there are limited studies on the examination of the relationship between these three skills with structural equation modeling, the model created offers a methodological innovation in terms of revealing the relationship between critical skills with concrete data. Thus, it is anticipated that it will contribute methodologically to similar studies and encourage its use in the literature in terms of examining the relationships between critical skills and other variables. In addition, it is expected that the results obtained will pave the way for studies examining the relationships between critical skills in teacher education in more detail. It is thought that the results of this research will contribute to the guidance of practices aimed at developing the critical skills of teacher candidates in teacher education programs and to focus more on the acquisition of these skills. It is expected that teachers who are raised more consciously and questioningly will contribute to an increase in the quality of education by integrating the critical skills they possess into the education process.

**Figure 1:**
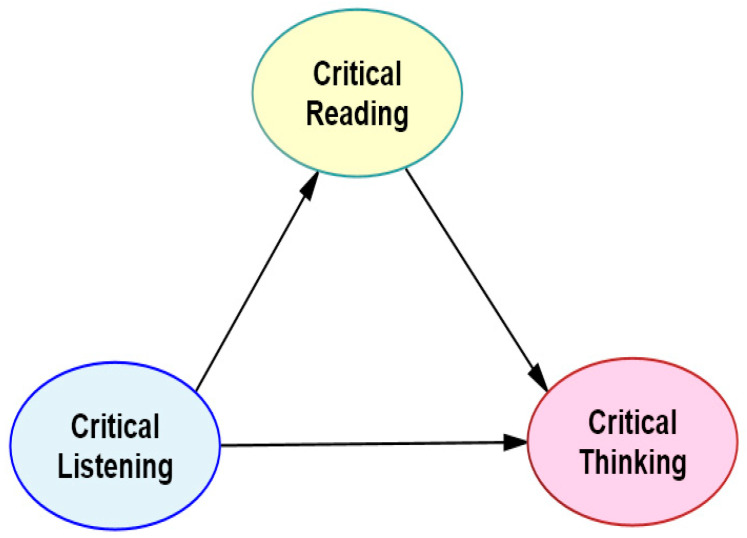
Study model.

The conceptual model of the relationship between these three variables, the theoretical foundations of which are discussed in the relevant literature review, based on existing studies, is presented in Figure 1.

Within the framework of the above model, this study aims to determine the interinfluence of pre-service teachers’ critical listening, critical reading, and critical thinking skills; how they predict each other; and their levels of explanation. The hypotheses proposed for testing in the model created for this purpose are as follows:

**H_1_:**  *Critical listening significantly and positively predicts critical thinking*.

**H_2_:**  *Critical listening significantly and positively predicts critical reading.*

**H_3_:**  *Critical reading significantly and positively predicts critical thinking.*

## 2. Materials and Methods

### 2.1. Research Model

This study examines the influence of Turkish teacher candidates’ critical reading and critical listening skills on their critical thinking skills and the relationships and explanation levels between these variables and was conducted using the correlational survey model, a relational survey model. This model determines the existence and/or degree of co-variation between two or more variables and explains the effect of the independent variable on the dependent variable (Cohen et al., 2000; Frankel & Wallen, 2006).

### 2.2. Study Group 

According to the statistics announced by the Council of Higher Education (YÖK), it was determined that the number of students studying in teacher education with subject specialization in state universities in Türkiye in the 2023–2024 academic year was 157,777 (YÖK, 2024). The universe of this research consists of 157,777 students studying at state universities in Turkey. Since it was difficult to reach all the students in the research population, a sampling selection was made, and teacher candidates studying in the Turkish language teaching department of a university in the north of Türkiye were selected from the universities within the scope of this research using the convenience sampling method (Büyüköztürk et al., 2016). The total number of teacher candidates studying in the Turkish language teaching department at this university was 223. In cases where the research universe is known, the sample size calculation formula is used to determine the sample size. With this formula, the sample size was calculated for the sample size selected from the research universe within the 95% confidence limits and with a 5% margin of error rate. As a result of this calculation, the sample size was found to be 141. The calculation is given below (Anderson, 1988; Newbold, 2005). For Turkish language teacher candidates:

$$n=\frac{223*1.96^{2}*0.5*0.5}{0.05^{2}\left(223-1\right)+1.96^{2}*0.5*0.5}=141\;\mathrm{teacher}\;\mathrm{candidates}$$

The G-Power program was used to determine the sample power. To calculate the sample size, the mean, standard deviation, and relationship levels of the groups obtained in previous studies or the ratios of the variables to each other must be known, from which the effect size can be calculated using these ratios and information. In this study, to decide how many people would be in the sample group, the study titled “Attitudes of Pre-Service Teachers towards Critical Listening (Kemiksiz, 2023)” published in 2023 was examined. As a result, the analyses made by the researcher in the study taken as a reference for this study were examined and the effect size was calculated as 0.6097007. In the light of this information, α = 0.05, 1 − β = 0.90 was taken, and the sample size was calculated as 116 people in total with an effect size of 0.6097007. When α = 0.05, 1 − β = 0.80 was taken, the sample size was calculated as 88 people in total with an effect size of 0.6097007. The screenshot of the protocol screens for G-power results is given below.

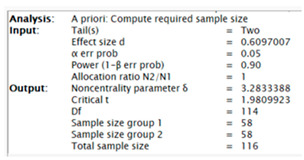

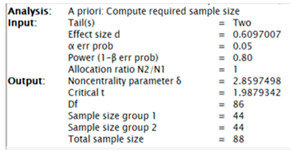
(Screenshot of α = 0.05, 1 − β = 0.90)(Screenshot of α = 0.05, 1 − β = 0.80)

In line with all these results, although the entire sample of this study was reached, 201 participants were determined when the outliers were removed. This result was determined to be higher than both the sample number in the G-Power power analysis and the result obtained from the known universe sample volume formula. As a result, the sample of this study consists of 201 teacher candidates studying in the Turkish language teaching department of a university in the north of Türkiye. The characteristics of the participants in the sample are given in Table 1.

According to Table 1, 24.9% (50) of the teacher candidates were in the first year, 27.4% (55) in the second year, 24.4% (49) in the third year, and 23.4% (47) in the fourth year; 33.8% (68) were male and 66.2% (133) were female. The age range of the participants varies between 18 and 27. 53% of the participants are between the ages of 18–20, 39% are between the ages of 21–23, and 9.95% are between the ages of 24–27.

The reason why Turkish teacher candidates were chosen as the study group of this research is that these teacher candidates were training to teach at the secondary school level. The basic stage in which critical skills are acquired in language teaching is the secondary school level, and at this stage, children pass from linguistic reading to the stage of acquiring critical reading, monitoring, listening, and thinking skills (10–12 years of age) (Sever, 2017). Since the teachers who undertake the main role in acquiring these skills at the secondary school level are Turkish teachers, the participants in this research were chosen from these teacher candidates. Thus, the current situation of Turkish teachers in the pre-service period was determined, and research was conducted on these participants to both provide them with higher quality education and to provide their students with these skills in the most competent way in their professional lives.

### 2.3. Data Collection Tools

The data were gathered through the Critical Listening Scale, the Critical Thinking Attitude Scale, the Critical Reading Self-Efficacy Perception Scale, and a personal information form.

#### 2.3.1. Personal Information Form

To determine the demographic variables of the participants in the study, a personal information form was used. Through this form, information about the class levels, ages, and genders of the Turkish teacher candidates was collected.

#### 2.3.2. Critical Reading Self-Efficacy Perception Scale

The Critical Reading Self-Efficacy Perception Scale, developed by Karadeniz (2014) to measure university students’ self-efficacy perceptions of critical reading, is a 5-point Likert-type (5 = strongly agree; 4 = agree; 3 = undecided; 2 = disagree; 1 = strongly disagree) measurement tool consisting of 33 items and five factors: “questioning”, “analysis”, “evaluation”, “finding similarities and differences”, and “making inferences”. The Cronbach’s alpha reliability coefficient for the entire scale is 0.94, and for the sub-factors, it is 0.87, 0.83, 0.84, 0.83, and 0.80, respectively. The confirmatory factor analysis (CFA) conducted on the scale show that the structural model’s fit indices (χ^2^/df = 2.04 (*p* < 0.01); RMSEA = 0.04; SRMR = 0.04; GFI = 0.91; AGFI = 0.90; CFI = 0.98; NFI = 0.97) indicate the model is acceptable.

The reliability and validity study of the Critical Reading Self-Efficacy Perception Scale was conducted for this study and these analyses are given below.

Confirmatory Factor Analysis: In order to determine whether the original factor structures of the scale were confirmed within the framework of this study, confirmatory factor analysis (CFA) was conducted and goodness of fit values (χ^2^/sd = 1.69 (*p* < 0.01); GFI = 0.80; CFI = 0.90; RMSEA = 0.05; SRMR = 0.05) were calculated. According to these results, it can be said that the goodness of fit index values of the model created with the four-factor structure of the scale are at an acceptable level (Hu & Bentler, 1999; Jöreskog & Sörbom, 1993; Kline, 2011; Sümer, 2000).

Reliability Analysis: As a result of the reliability analysis conducted on the scale, the Cronbach’s alpha internal consistency coefficient of the scale consisting of 33 items and five factors was calculated as 0.85 for questioning, 0.86 for analysis, 0.86 for evaluation, 0.86 for finding similarities and differences, 0.86 for making inferences, and 0.86 for the total of the scale.

When the literature was examined, since there was no measurement tool regarding the critical reading attitudes of teacher candidates, the critical reading self-efficacy perception scale was used in this study. Other measurement tools regarding critical reading were also examined in the relevant literature, and since this measurement tool is the most competent measurement tool that fully covers the critical reading skills of the sub-factors of “questioning”, “analyzing”, “evaluating”, “finding similarities and differences”, and “making inferences”, it was decided to use this measurement tool. Critical reading is a reading method that includes cognitive processes such as wondering, questioning, criticizing, and self-criticism, which require thinking about what is read, combining thoughts, and evaluating (DeVoogd, 2008; Flemming, 2011; Özdemir, 2011). It requires a process that includes comparing the information conveyed by the author, instead of accepting it as it is, and making a judgment (Aşılıoğlu, 2008). In addition to including these cognitive processes, this measurement tool was used, as it was concluded that it is a multi-dimensional measurement tool that also includes the variables of the critical thinking process in the reading process.

#### 2.3.3. Critical Thinking Attitude Scale

The Critical Thinking Attitude Scale, developed by Özelçi (2012) to determine teacher candidates’ attitudes towards critical thinking, is a 5-point Likert-type (5 = strongly agree; 4 = agree; 3 = undecided; 2 = disagree; 1 = strongly disagree) measurement tool consisting of 19 items and five factors: “willingness to gather information”, “self-regulation”, “making inferences”, “evidence-based decision-making”, and “openness to seeking reasons”. The Cronbach’s alpha reliability coefficient for the sub-factors is 0.70, 0.64, 0.52, 0.54, and 0.56, respectively. The confirmatory factor analysis (CFA) results confirmed the five-factor structure of the scale. The structural model’s fit indices (χ^2^ = 235.19, df/χ^2^ = 0.60, RMSEA = 0.04, NNFI = 0.80; CFI = 0.91; AGFI = 0.92) indicate the model is acceptable. 

The reliability and validity study of the Critical Thinking Attitude Scale was conducted for this study and these analyses are given below.

Confirmatory Factor Analysis: In order to determine whether the factor structures of the original form of the scale were confirmed within the framework of this study, confirmatory factor analysis (CFA) was conducted and goodness of fit values (χ^2^/sd = 1.48 (*p* < 0.01); GFI = 0.91; CFI = 0.91: RMSEA = 0.04; SRMR = 0.06) were calculated. According to these results, it can be said that the goodness of fit index values of the model created with the four-factor structure of the scale are at an acceptable level (Hu & Bentler, 1999; Jöreskog & Sörbom, 1993; Kline, 2011; Sümer, 2000).

Reliability Analysis: As a result of the reliability analysis conducted on the scale, it was confirmed that the scale consisted of 19 items and five factors. The Cronbach Alpha internal consistency coefficient of the scale was calculated as 0.86 for the sub-dimensions of the scale, which are willingness to gather information, 0.87 for self-regulation, 0.87 for making inferences, 0.87 for evidence-based decision making, 0.87 for openness to reason-seeking, and 0.87 for the total scale.

One of the basic components of critical thinking is to develop an attitude towards critical thinking. The three basic elements that constitute the critical attitude are open-mindedness, sincerity, and confronting the results, and these three characteristics are the elements that provide a positive attitude towards critical thinking. For an individual to be defined as a good thinker, they need to be willing to search for meaning, gather information, reconsider solutions and to associate these skills with metacognitive skills. At this point, it can be said that the basic element that ensures a high critical thinking attitude is the competencies related to the organization among metacognitive skills (Özelçi, 2012). When the literature is examined, there are a limited number of measurement tools that examine the critical thinking attitudes of prospective teachers. In addition, this measurement tool was preferred for this study because it is the only measurement tool for prospective teachers who use the factors related to metacognitive skills such as “willingness to gather information”, “self-regulation”, “making inferences”, “evidence-based decision making”, and “openness to seeking reasons” together, which will provide information gathering and transforming this information.

#### 2.3.4. Critical Listening Scale

The Critical Listening Scale, developed by Taşkın (2017) to determine pre-service teacher attitudes while listening, is a 5-point Likert-type (5 = strongly agree; 4 = agree; 3 = undecided; 2 = disagree; 1 = strongly disagree) measurement tool consisting of 20 items and three factors: “comprehension”, “analysis”, and “evaluation” of what is listened to. The confirmatory factor analysis (CFA) conducted on the scale show that the structural model’s fit indices (χ^2^/df = 1.57 (*p* < 0.01); RMSEA = 0.05; SRMR = 0.06; GFI = 0.90; CFI = 0.90) indicate the model is acceptable. 

The reliability and validity study of the Critical Listening Attitude Scale was conducted for this study. and these analyses are given below.

Confirmatory Factor Analysis: In order to determine whether the factor structures of the original form of the scale were confirmed within the framework of this study, confirmatory factor analysis (CFA) was conducted and goodness of fit values (χ^2^/sd = 1.63 (*p* < 0.01); GFI = 0.89; CFI = 0.87; RMSEA = 0.05; SRMR = 0.06) were calculated. According to these results, it can be said that the goodness of fit index values of the model created with the four-factor structure of the scale are at an acceptable level (Hu & Bentler, 1999; Jöreskog & Sörbom, 1993; Kline, 2011; Sümer, 2000).

Reliability Analysis: As a result of the reliability analysis conducted on the scale, it was confirmed that it consisted of 20 items and three factors. The Cronbach Alpha internal consistency coefficient of the scale was calculated as 0.88 in the sub-dimensions of the scale, “making sense of what is listened to (comprehension)”, 0.86 in the “dimension of questioning (analysis)” what is listened to, 0.87 in the “dimension of interpreting (evaluating)” what is listened to, and 0.85 in total.

As a result of the literature review, it was determined that there was a limited number of critical listening attitude scales for teacher candidates. The main reason for using this measurement tool among the relevant measurement tools is that it is the only scale in the literature that includes the main elements of criticality in the listening process, namely, questioning, analysis, and evaluation stages. The reason for considering these sub-factors in the measurement tool is that these sub-factors include the main elements of critical listening, which enable individuals to thoroughly analyze and evaluate the information presented before accepting or rejecting it (Bourdeaud’hui, 2020). Since critical listening includes the stages of understanding, evaluation, and critically evaluating and interpreting, it was determined that this measurement tool is a tool that reflects attitudes towards the skills targeted in critical listening (Baharman et al., 2020), and it was decided to use this measurement tool.

### 2.4. Data Collection

In this study, data were collected using the Critical Listening Attitude Scale, Critical Thinking Attitude Scale, Critical Reading Self-Efficacy Perception Scale, and a personal information form. These measurement tools were applied to all prospective teachers studying in the Turkish language teaching department of a university in the north of Türkiye. After the planning for the application of the scales, all scales were applied as paper-based at the same class hour at each grade level. It took 60 min to complete the scales.

### 2.5. Data Analysis

In this study, SPSS 23 and AMOS 22.0 software were used to analyze the relationship pattern between the variables of critical listening, critical thinking, and critical reading, and descriptive statistics and measures of central tendency and dispersion (frequency, percentage, mean, kurtosis, and skewness) were calculated for demographic variables. Structural equation modeling (SEM) was used to calculate descriptive statistics for variables and to evaluate the relationships between variables in this study. Structural equation modeling (SEM), which is a statistical method used to test the relationships between observed and latent variables, allows for the measurement of direct and indirect relationships by taking into account the measurement errors in the observed variables and the relationships between the errors with a single model. Incorporating multiple statistical methods, SEM allows for the analysis of complex relationships in cases where there is more than one dependent variable (Jöreskog & Sörbom, 1993). 

Before the SEM analysis, a two-stage method widely used in this analysis was used to evaluate whether the data supported the model (Jöreskog & Sörbom, 1993; Meydan & Şeşen, 2011). First, a preliminary examination of the applied scales was performed, it was determined that there were no missing or unreliable scales, and then the scales were coded and classified by numbering. In the first stage, CFAs were applied to the scales to determine the factor structures, validity, and reliability of the scales in the model. The CFA results for each scale were given in the sections where information about the relevant scales was explained, and it was revealed that the scales showed a good fit with the obtained data and that the factor structures in their original form were also confirmed for this study. Within the framework of the preliminary analyses, the missing data rate in the data set was first examined and values reflecting the mean of the series were assigned for the missing values observed in the data set. Then, to determine the extreme values (outliers) in the data set, the scores were converted to standardized z-scores and because of the analysis, twenty-two extreme values were detected and removed from the data set. After this transformation, the skewness and kurtosis values of the variables were calculated so that univariate normality could be assumed for the data (Chou & Bentler, 1995).

In the second stage, the relationships between the variables in the model were examined. Before starting the SEM analysis, the assumptions regarding this analysis were checked. The sample size and multivariate normality assumptions required for this analysis were tested. It is recommended that the sample size recommended for SEM should consist of at least 200 people (Kline, 2011). The skewness and kurtosis values for each variable were calculated for univariate normality, which is a prerequisite for meeting the multivariate normality assumption (Kline, 2011). In examining the multivariate normality assumption, Mardia’s normalized multivariate kurtosis coefficient was calculated (Raykov & Marcoulides, 2008). After the assumptions regarding the data set were met, the compatibility of the model with the data set was examined. In the analyses related to the estimation of parameters in SEM, the maximum likelihood technique was preferred. As a result of the analysis, χ^2^/df, CFI, GFI, TLI, NFI, IFI, RMSEA, and SRMR fit indices were used in the assessments related to the fit of the model. The fit levels of the fit indices to the model because of the analyses are given in Table 2 (Baumgartner & Homburg, 1996; Bentler, 1990; Hu & Bentler, 1999; Kline, 2011; Klem, 2000; Marsh et al., 2004).

## 3. Findings

The findings are presented in two sections: the first section includes descriptive statistics while the second includes findings related to the variables in the structural equation modeling, the predictions of the relationships between these variables, the explanation rates of the variables, and the model fits.

### 3.1. Descriptive Statistics Related to Model Variables

Descriptive statistics for the variables included in the research model are presented in Table 3.

According to Table 3, the arithmetic means for all variables included in the research model range between 53.88 and 7.37 points. For the assumption of univariate normality to be valid, the skewness and kurtosis values of the variables should be less than |3.0| and |10.0|, respectively (Kline, 2011). Accordingly, the skewness values for the variables range from −0.324 to −0.001, and the kurtosis values range from 0.055 to −0.618. These findings indicate that univariate normality is satisfied for the data. For multivariate normality, Mardia’s normalized multivariate kurtosis coefficient was calculated to be 10.77. The critical value for multivariate normality was calculated as *p*(*p* + 2) (*p*: number of observed variables) according to the equation proposed by Raykov and Marcoulides (2008), resulting in a value of 195. According to Raykov and Marcoulides (2008), the critical value from the equation should be greater than the kurtosis coefficient for multivariate normality to be satisfied. As the value obtained from the equation (195) is greater than the multivariate kurtosis coefficient (10.77), the assumption of multivariate normality is considered satisfied.

### 3.2. Findings Related to the Measurement Model

**Figure 2:**
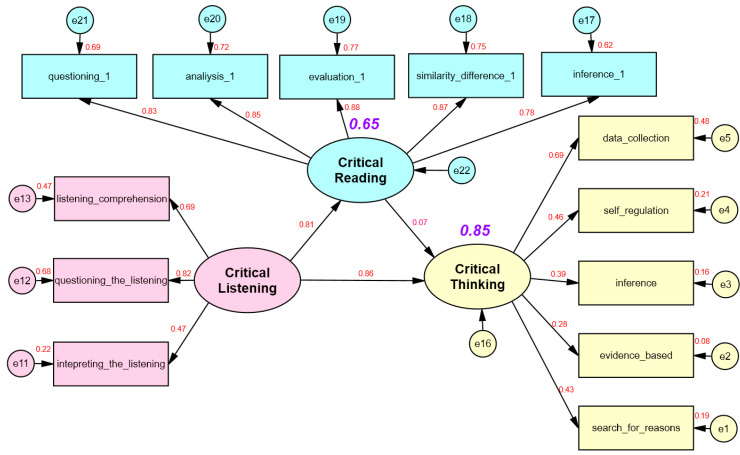
The effect of critical listening and critical reading on critical thinking.

The results of testing the model, which was created to examine the relationship between critical listening, critical thinking, and critical reading and to determine the predictive power of these variables on critical thinking, are presented in Figure 2. 

Based on the structural equation modeling results, the goodness of fit indices (χ^2^/df = 2.17; GFI = 0.91; CFI = 0.94; TLI = 0.93; NFI = 0.90; IFI = 0.94; RMSEA = 0.07; and SRMR = 0.05) can be considered acceptable. In the structural model test, the factor loadings of the latent variable of critical listening ranged from 0.47 to 0.82, the factor loadings of the latent variable of critical reading ranged from 0.78 to 0.88, and the factor loadings of the latent variable of critical thinking ranged from 0.28 to 0.69. Standardized regression weight results are presented in Table 4.

According to Table 4, two of the three hypotheses evaluated within the model framework were supported by the data, and one hypothesis was rejected. In the model, it was seen that critical listening had a positive and significant effect on critical thinking (*β* = 0.86, *p* < 0.01), and the hypothesis “H_1_: Critical listening predicts critical thinking positively and significantly” was accepted. It was seen that critical listening had a positive and significant effect on critical reading (*β* = 0.81, *p* < 0.01), and the hypothesis “H_2_: Critical listening predicts critical reading positively and significantly” was accepted. It was seen that the effect of critical reading on critical thinking was not significant (*β* = 0.07, *p* > 0.05), and the hypothesis “H_3_: Critical reading predicts critical thinking positively and significantly” was rejected. Standardized direct, indirect, and total effect sizes are presented in Table 5.

According to Table 5, the variables in the model explain 65% of the total variance in critical reading, while critical listening and critical reading together explain 85% of the total variance in critical thinking. In another parlance, critical listening indirectly affected critical thinking through critical reading, and the indirect effect of critical listening on critical thinking through critical reading was significant. The indirect effect of critical reading on critical thinking was not significant. Thus, critical reading had a significant impact on critical thinking only through the mediation of critical listening.

## 4. Discussion and Implication

This study aimed to examine the relationship between critical listening, critical reading, and critical thinking and to determine the predictive effect of these variables on critical thinking, and the hypotheses created were examined through structural equation modeling. Three hypotheses were tested within the scope of the research model.

As a result of testing the first hypothesis of this study, it was determined that critical listening predicted critical thinking positively and significantly (H_1_). In addition, it can be said that critical listening has a direct and high effect on critical thinking, and that critical thinking also develops depending on the development of critical listening. Similar studies support this result and reveal the effect of critical listening on developing critical thinking (Aghaei & Rad, 2018; Gunawan et al., 2023). Indeed, in the study of Hyytinen et al. (2021), it was determined that critical listening studies conducted to determine the errors in the presentation of a subject developed critical thinking. In the study of Smialek & Boburka (2006), it was revealed that critical thinking and critical listening are related skills. In the literature, critical listening is accepted as one of the basic foundations of critical thinking (Aslan, 2021). As a result of this study, it can be said that critical listening has an effect on critical reading, based on the confirmation of the hypothesis that critical listening is a significant predictor of critical thinking. In related studies, the effect of the use of original listening materials on the effect of critical listening on critical thinking should not be ignored. As a matter of fact, these original listening materials are also an effective factor in the development of critical thinking (Harida, 2023; Puspita & Amelia, 2020), critical listening (Harida, 2023), and listening comprehension (Puspita & Amelia, 2020) skills. In addition to the effect of critical listening on listening skills (Gunawan et al., 2023), studies revealing the positive and strong relationship between critical thinking and listening comprehension (Aghaei & Rad, 2018; Elekaei et al., 2016; Etemadfar et al., 2020; Nour Mohammadi & Zare, 2015) also draw attention to the importance of listening skills in the development of critical thinking skills. Based on these results, it can be said that there is a need to integrate critical listening, critical thinking and listening. Because in order for an individual to discover the truths in the messages he/she receives, he/she must first have listening and critical listening skills (Bourdeaud’hui et al., 2021). Based on the research results in the literature and the verification of the hypothesis of this research, it can be said that critical listening and listening are effective in the development of critical thinking and that using these two variables together can influence developing critical thinking.

When the second hypothesis of this study was tested, it was determined that critical listening predicted critical reading positively and significantly (H_2_). As a result of this study, it can be said that critical listening explained 65% of the variance related to critical reading; critical listening has a direct and very high effect on critical reading. Based on this finding, it can be said that as critical listening improves, critical reading can also improve. As a result of the examinations, no research was found that directly examined the relationship between critical listening and critical reading. However, as Tubail (2015) stated, critical listening—which includes various levels such as reception, discrimination, comprehension, analysis, interpretation, realization, inference, evaluation, judgment, and reaction—positively contributes to the critical reception (understanding) of the text. Studies show that activities based on critical listening facilitate the determination of the subject and main idea of the text listened to and develop text comprehension skills (Azizoğlu, 2020; Çarkıt, 2018). In Arslan’s (2022) research, it was determined that critical reading levels have a positive effect on the levels of using listening/watching strategies. The results obtained from the studies in the literature and in this research suggest that the coordinated conduct of critical listening studies and critical reading studies may influence the development of critical reading.

When the third hypothesis of this study was tested, it was determined that the effect of critical reading on critical thinking was insignificant and therefore critical reading did not predict critical thinking positively and significantly, and this hypothesis was rejected (H_3_). However, in the study conducted by Akdan (2016) with Turkish teacher candidates, it was determined that there was a positive, moderately significant relationship between critical thinking and critical reading; critical thinking explained 19% of critical reading. Studies have shown that there is a high (Hidayati et al., 2020) and moderate (Tous et al., 2015) positive, significant relationship between critical reading and critical thinking, and that critical reading activities improve critical thinking (Oroujlou & Sadeghi, 2022; Türkben & Karaman, 2022; Yıldırım & Söylemez, 2018). In addition to these studies, there are various studies examining the effect of critical thinking on critical reading. In related studies, it has been determined that critical thinking is a significant predictor of critical reading (Aghajani & Gholamrezapour, 2019) and has a significant effect on the development of critical reading (R. Paul & Elder, 2006; Abdel Halim, 2011). Since reading and thinking are integrated skills, it can be said that the active use of thinking processes directly affects the reading process and, therefore, the fluent application of critical thinking skills is necessary for the active use of critical reading (Zin et al., 2014). This is because the goal of basic language skills is to increase the individual’s thinking power and enable them to reason effectively (Aghajani & Gholamrezapour, 2019). Indeed, studies also support the relationship between these two variables (Abdel Halim, 2011; Larsson, 2017; Y. H. Lee, 2015; Mayfield, 2014; Medina & Pilonieta, 2006). Although most studies in the literature have shown that there is a significant relationship between critical thinking and critical reading, and that critical reading studies influence developing critical thinking, it can be said that this effect was not revealed in this study. Similarly, Din’s (2020) study revealed that university students’ critical thinking skills could not be reflected in the critical reading process. Tufan’s (2008) study found that the critical thinking levels and reading habits of prospective teachers, and Şen’s (2009) study found that the reading frequency of prospective Turkish teachers did not have a significant effect on their critical thinking attitudes. When the results of these studies are examined, it can be said that the interaction between critical thinking and reading could not be established.

As a result of this research, contrary to the studies confirming the relationship between critical reading and critical thinking in the literature, it was determined that critical reading does not have a significant effect on critical thinking. The reasons for this situation may include the fact that Turkish teacher candidates have not received training in critical reading or critical thinking, the teaching methodology used in their education, and the type of reading material used in reading studies not having characteristics that develop critical reading and critical thinking. The reasons why students are deprived of critical thinking include teachers’ emphasis on transferring information, and the uniformity that causes students to accept all information without questioning by giving unnecessary and excessive information (Glasser, 2000). In addition, students’ mindset and inability to think independently; the effect of the physical environment and the large number of students; prejudices, prejudices, blind devotion to ideologies, and difficulties in planning due to the time taken for critical thinking activities can be listed (Ay & Akgöl, 2008; Hotaman, 2020; Şahinel, 2007; Sünbül, 2011). The reasons why teachers cannot acquire critical thinking and critical reading skills include the existence of an exam-centered education system, teachers’ focus on exams, and concerns about completing curriculum programs (Akdan, 2016). In addition, teachers’ critical reading and critical thinking skills have not developed during the pre-service education process, teachers do not give the necessary importance to these skills, they adopt a traditional education approach instead of a critical approach in education, they are given information that is not based on questioning and judgment, and they do not believe in the necessity of education, courses, activities, etc., related to critical reading and thinking (Kökdemir, 2003). In addition, when the current undergraduate education programs of the Turkish teacher candidates who are the participants of this study are examined, it is seen that independent courses on critical reading and critical thinking have been put into practice since the 2018–2019 academic year. With the changes made in the Turkish Language Teaching Undergraduate Program, the “Critical Reading Course” has taken its place in the field education elective courses category, and the “Critical and Analytical Thinking Course” has taken its place in the professional knowledge elective courses category (YÖK, 2019). These courses are in the elective courses category in the relevant undergraduate program, and it is known that not all teacher candidates take these courses. Based on these data, the fact that Turkish teacher candidates have not received compulsory training on critical reading and critical thinking, and the fact that these courses have been added to the undergraduate program recently, can be considered as a factor in the teacher candidates not having the necessary accumulation in terms of these critical reading and critical thinking skills. In addition, the limitation of the measurement tool used in this study may have also been effective in revealing this result, as the attitude scale was used to measure critical thinking, and the self-efficacy perception scale was used to measure critical reading. Self-efficacy perception is the belief of an individual about his/her own level of competence regarding the capacity to organize the activities required to demonstrate a certain performance and to carry them out them successfully, and it includes a general ability that complements cognitive, social, and behavioral skills (Bandura, 1982). Attitude is the tendency to react to a situation (Bordens & Horowitz, 2002). Attitudes, which are based on the individual’s emotions, beliefs, and values and have an integrative role on their behaviors (Crano & Prislin, 2006), are the forces that manage and guide the behaviors of individuals (Ajzen, 2014). As a result of the research, the reason why critical reading does not affect critical thinking can be shown as the inconsistency between the attitudes of the prospective teachers and their self-efficacy beliefs. In other words, this result may be due to the difference between the self-efficacy perceptions of the prospective teachers and their attitudes that have the power to take action.

As a result of this research examining the effects of critical listening and critical reading on critical thinking, it can be said that these two variables explain 85% of critical thinking, and that critical listening has a direct and positive effect on critical thinking, whereas the direct effect of critical reading is insignificant, so the main predictor of critical thinking is critical listening. In Kamçı’s (2024) research with prospective teachers, it was determined that critical reading is a significant positive predictor of critical listening and thinking. In Dewati’s (2020) research, the effect of critical thinking on reading and listening skills was revealed, and these two variables were evaluated through critical thinking. When the views in theoretical studies revealing the effect and importance of these three variables on each other in the literature are examined, it is stated that critical listening interacts with critical reading and critical thinking (Erkek & Batur, 2020; Mamman-Muhammad, 2018), and critical reading and critical listening are among the cognitive strategies of critical thinking (R. W. Paul, 1990). It can be said that these views draw attention to the effect of critical reading and critical listening on the cognitive processes of critical thinking. Based on the effect of critical listening on critical thinking (Gunawan et al., 2023) and the results of this research hypothesis, it can be said that including critical listening, which is a skill that contributes to the development of language skills, in critical thinking studies (Gunawan et al., 2023) can improve critical thinking. In addition, considering that critical listening has a high direct effect and explanation rate on critical reading in this study, it can be said that carrying out critical reading activities together with critical listening can improve critical reading skills. Based on the effect of critical listening on critical thinking, it is predicted that including critical listening in critical thinking activities will improve critical thinking.

## 5. Limitations

The results of the current study should be evaluated by considering some limitations. This study examined the effects of critical reading and critical listening on critical thinking and the relationship between these variables, and this study was conducted only from the perspective of the teacher candidate. In future studies, the validity of this model can be tested in different samples (primary school, middle school, high school, etc.) and the interaction of these variables can be examined. This study is limited to the variables of critical listening, critical reading, and critical thinking, and it can be suggested that the model be expanded with other variables in future studies to examine the effects of the variables affecting critical thinking. As a result of the literature review on the measurement tools, only the self-efficacy perception scale regarding critical reading was reached. Since the measurement tool is limited to the perceptions of teacher candidates regarding critical reading, the results obtained should be evaluated in this context. Another limitation of this study is that the results obtained are based on cross-sectional data and do not allow for inferences to be made regarding the cause–effect relationship. For this reason, it can be recommended that longitudinal studies examining the relationship between the variables of critical reading, critical thinking, and critical listening be conducted to make inferences based on the possible cause–effect relationship between these variables.

## 6. Conclusions and Recommendations

In this study, where the effects of critical listening and critical reading on critical thinking and the relationship between these variables were examined, a conceptual model created based on the literature was verified; two of the three hypotheses were accepted, and one was rejected. As a result of this study, it can be said that critical listening has a very high effect on critical thinking, and as critical listening develops, critical thinking develops, and therefore, including critical listening in critical thinking studies will contribute to the development of this thinking skill. Considering the high effect of critical listening on critical reading and the explanation rate, it is thought that conducting critical reading and critical listening studies together will contribute to the development of critical reading. Considering the insignificance of the effect of critical reading on critical thinking, it can be said that critical thinking and critical listening do not affect critical thinking together, and therefore, there are other factors that affect the relationship between critical thinking and critical reading. To determine what these factors are, this result can be examined in detail with action research or qualitative research that will allow for an in-depth examination of this relationship. In new conceptual models to be created regarding critical thinking and critical listening, the mediating role of critical reading in the interaction between critical listening and critical thinking can be examined. Under current conditions, critical reading is expected to affect critical thinking. It can be said that including critical listening in new models to be created based on the effect of critical listening on critical thinking can create positive effects and that other variables that affect the development of critical thinking can be determined by adding other variables to the model and thus contribute to the development of critical thinking skills.

## Figures and Tables

**Table 1:** Characteristics of the study group.

Features		Distribution	
		*f*	%
Gender	Female	133	66.2
	Male	68	33.8
Age	18–20	107	53.23
	21–23	80	39.80
	24–27	20	9.95
Class	1st year	50	27.4
	2nd year	55	24.4
	3rd year	49	24.4
	4th year	47	23.4

**Table 2:** Fit indices for structural equation models.

Fit Indices	Acceptable Fit
χ^2^/sd	2 < χ^2^/sd ≤ 5
RMSEA	0.05 < RMSEA ≤ 0.08
SRMR	0.05 ≤ SRMR ≤ 0.10
IFI	0.90 ≤ IFI < 0.95
TLI	0.90 ≤ TLI < 0.95
CFI	0.90 ≤ CFI < 0.95
GFI	0.90 ≤ GFI < 0.95
NFI	0.90 ≤ NFI < 0.95

**Table 3:** Descriptive statistics for measurement items.

Factor	Min–Max	M	SD	Skewness	Kurtosis
Willingness to gather information	10–20	15.57	2.28	−0.22	−0.18
Self-regulation	12–25	19.28	2.52	−0.10	0.24
Making inferences	8–15	13.39	1.71	−0.87	0.05
Evidence-based decision making	3–15	10.36	3.06	−0.24	−0.61
Openness to seeking reasons	8–20	16.88	3.05	−0.97	0.38
Listening comprehension	27–53	40.06	4.60	0.21	−0.18
Listening questioning	12–25	19.75	2.80	−0.28	0.07
Interpreting the listening	8–20	15.63	2.17	−0.32	0.33
Inquiry	23–45	15.50	4.38	−0.00	0.15
Analysis	18–35	19.28	3.39	−0.03	0.12
Evaluation	14–30	13.39	3.51	−0.32	0.35
Finding similarities and differences	12–25	10.36	2.74	−0.22	0.10
Making inferences	12–25	16.88	2.72	0.11	0.05

**Table 4:** Standardized regression weights results.

Path	Path Coefficient *(β*)	Standardized Estimate	Standard Error (S.E.)	Critical Ratio (C.R.)	Significance Value (*p*)
Critical listening → Critical thinking	0.86	1.109	0.33	3.31	***
Critical listening → Critical reading	0.81	1.67	0.28	5.82	***
Critical reading → Critical thinking	0.07	0.44	0.11	0.39	0.69

*** significant in *p* < 0.01 value.

**Table 5:** Standardized direct, indirect, and total effect sizes.

Predicted	R^2^	Predictor	Standardized Estimates		
			Direct	Indirect	Total
Critical reading	0.65	Critical listening	0.80	---	0.80
Critical thinking	0.85	Critical listening	0.86	---	0.92
		Critical reading	0.07	---	0.07

## Data Availability

The original contributions presented in this study are included in the article; further inquiries can be directed to the corresponding author.
